# miRNAs and Genes Involved in the Interplay between Ocular Hypertension and Primary Open-Angle Glaucoma. Oxidative Stress, Inflammation, and Apoptosis Networks

**DOI:** 10.3390/jcm10112227

**Published:** 2021-05-21

**Authors:** Jorge Raga-Cervera, Jose M. Bolarin, Jose M. Millan, Jose J. Garcia-Medina, Laia Pedrola, Javier Abellán-Abenza, Mar Valero-Vello, Silvia M. Sanz-González, José E. O’Connor, David Galarreta-Mira, Elena Bendala-Tufanisco, Aloma Mayordomo-Febrer, Maria D. Pinazo-Durán, Vicente Zanón-Moreno

**Affiliations:** 1 Hospital of Manises, 46940 Valencia, Spain; joracer22@gmail.com; 2 Technological Centre of Information and Communication Technologies (CENTIC), 30100 Murcia, Spain; josemiguel.bolarin@centic.es (J.M.B.); javier.abellan@centic.es (J.A.-A.); 3 Sequencing Service at the University and Polytechnic Hospital La Fe, 46026 Valencia, Spain; millan_jos@gva.es (J.M.M.); sequencing_service@iislafe.es (L.P.); 4 Ophthalmic Research Unit “Santiago Grisolía”/FISABIO, 46017 Valencia, Spain; jj.garciamedina@um.es (J.J.G.-M.); vavema@alumni.uv.es (M.V.-V.); dolores.pinazo@uv.es (M.D.P.-D.); vczanon@universidadviu.com (V.Z.-M.); 5 Department of Ophthalmology, General University Hospital “Morales Meseguer”, 30007 Murcia, Spain; 6 Department of Ophthalmology and Optometry, University of Murcia, 30120 Murcia, Spain; 7 Spanish Net of Ophthalmic Research OFTARED RD16/0008/0022, Institute of Health Carlos III, 28029 Madrid, Spain; elena.bendala@uch.ceu.es (E.B.-T.); aloma.mayordomo@uch.ceu.es (A.M.-F.); 8 Cellular and Molecular Ophthalmobiology Group, Department of Surgery, Faculty of Medicine and Odontology, University of Valencia, 46010 Valencia, Spain; 9 Laboratory of Cytomics, Joint Research Unit Principe Felipe Research Center and University of Valencia, 46010 Valencia, Spain; jose.e.oconnor@uv.es; 10 University Clinic Hospital of Valladolid, 47003 Valladolid, Spain; dgalarreta@saludcastillayleon.es; 11 Mixed Research Unit for Visual Health and Veterinary Ophthalmology CEU/FISABIO, 46020 Valencia, Spain; 12 Physiology Department, Faculty of Health Sciences, CEU University, Alfara del Patriarca, 46115 Valencia, Spain; 13 Animal Medicine and Surgery Department, Veterinary Medicine Faculty, CEU University, Alfara del Patriarca, 46115 Valencia, Spain; 14 Faculty of Health Sciences, Valencian International University, 46002 Valencia, Spain

**Keywords:** ocular hypertension, glaucoma, tears, miRNAs, next generation sequencing, biomarkers, genes, signaling pathways, oxidative stress, inflammation, apoptosis, neurodegeneration

## Abstract

Glaucoma has no cure and is a sight-threatening neurodegenerative disease affecting more than 100 million people worldwide, with primary open angle glaucoma (POAG) being the most globally prevalent glaucoma clinical type. Regulation of gene expression and gene networks, and its multifactorial pathways involved in glaucoma disease are landmarks for ophthalmic research. MicroRNAs (miRNAs/miRs) are small endogenous non-coding, single-stranded RNA molecules (18–22 nucleotides) that regulate gene expression. An analytical, observational, case-control study was performed in 42 patients of both sexes, aged 50 to 80 years, which were classified according to: (1) suffering from ocular hypertension (OHT) but no glaucomatous neurodegeneration (ND) such as the OHT group, or (2) have been diagnosed of POAG such as the POAG group. Participants were interviewed for obtaining sociodemographic and personal/familial records, clinically examined, and their tear samples were collected and frozen at 80 °C until processing for molecular-genetic assays. Tear RNA extraction, libraries construction, and next generation sequencing were performed. Here, we demonstrated, for the first time, the differential expression profiling of eight miRNAs when comparing tears from the OHT versus the POAG groups: the miR-26b-5p, miR-152-3p, miR-30e-5p, miR-125b-2-5p, miR-224-5p, miR-151a-3p, miR-1307-3p, and the miR-27a-3p. Gene information was set up from the DIANA-TarBase v7, DIANA-microT-CDS, and TargetScan v7.1 databases. To build a network of metabolic pathways, only genes appearing in at least four of the following databases: DisGeNet, GeneDistiller, MalaCards, OMIM PCAN, UniProt, and GO were considered. We propose miRNAs and their target genes/signaling pathways as candidates for a better understanding of the molecular-genetic bases of glaucoma and, in this way, to gain knowledge to achieve optimal diagnosis strategies for properly identifying HTO at higher risk of glaucoma ND. Further research is needed to validate these miRNAs to discern the potential role as biomarkers involved in oxidative stress, immune response, and apoptosis for the diagnosis and/or prognosis of OHT and the prevention of glaucoma ND.

## 1. Introduction

Glaucoma is a neurodegenerative disease and a leading cause of irreversible blindness, affecting over 60 million people worldwide [1]. The number of people with glaucoma will increase to 111.8 million by 2040 [2]. These estimates are important in guiding the designs of glaucoma screening, diagnosis and treatment, research milestones, and related public health strategies.

Primary open-angle glaucoma (POAG) is the most prevalent type of glaucoma, typically characterized by adult onset, chronic intraocular pressure (IOP) elevation, IOP-dependent progressive apoptotic retinal ganglion cell (RGC) death, and visual field loss [1,2]. Main clinical features of glaucomatous optic nerve degeneration include the following: optic disc deepening, papillary hemorrhages, and specific defects of the retinal nerve fiber layer (RNFL) [3].

Diagnosis of POAG underlies a variety of clinical hallmarks such as the IOP elevation (the major risk factor) and the optic nerve head changes, as reflected by the structural/functional imaging techniques to appropriately establish the glaucoma stage [4,5,6]. Some decades ago, the term ocular hypertension (OHT) arose to catalogue the eyes with elevated IOP but not displaying optic disc damage or altered visual field. In 1977, Shaffer warned professionals about the false sense of security that may involve the managing of these patients that only have OHT, but no signs of neurodegeneration (ND) [7], suggesting that the term glaucoma suspects is preferred to ocular hypertensives. Glaucoma suspects define the group of patients with increased IOP and borderline optic discs (mild-to-moderate alterations), RNFL anomalies and/or visual field changes as well as glaucoma family history and the occurrence of other POAG risk factors. These patients have a higher risk of undergoing optic nerve degeneration (OND) than the normal population [8]. Therefore, classic and emerging technologies are essential for the early detection of POAG damage, but the identification of glaucomatous pre-perimetric changes continues to be a challenging issue for ophthalmologists and researchers. As the elevated IOP is the main risk factor, current knowledge on the etiopathogenic mechanisms for HTO and POAG remains incomplete. Among the cellular and molecular processes underlying these diseases, the following have largely been considered: oxidative/nitrosative stress, mitochondrial failure, inflammation and immune response, autophagy/mitophagy, apoptosis, neurotoxicity, ND, etc. [3,4,5,6,7,8,9]. It is imperative that the above processes are individually and integrally addressed.

The regulation of gene expression and gene networks as well as the multifactorial pathways involved in glaucoma disease are high-priority landmarks for ophthalmic research. Recent data have pinpointed potentially interesting routes to mediate glaucomatous RGC dysfunction [9]. Meanwhile, hypotensive therapy (medical, laser, surgical) is the only way to fight against the elevated IOP [10,11]. Despite experimental advances in neuroprotection [12,13,14], there is no definitive cure for glaucoma ND. Despite great advances in glaucoma, there is still no reliable biomarker that can pre-clinically identify subjects at risk of POAG initiation and progression. Molecular-genetic diagnostic challenges for POAG are needed to complete the current knowledge in disease pathogenesis as well as to design new diagnostic and therapeutic strategies gathered under the recently proposed term glaucoma theranostics [15] for better eye care.

MicroRNAs (miRNAs) are a family of small endogenous non-coding, single-stranded RNA molecules (18–22 nucleotides) that regulate gene expression by either inhibiting mRNA translation or by degrading mRNA [16,17]. miRNAs are involved in the pathological processes of numerous diseases including eye pathologies [18,19]. Specific miRNAs have also been proposed to regulate IOP [20,21] as well as for use as noninvasive biomarkers for the diagnosis of glaucoma [22,23,24]. Our group has been widely utilizing tear samples for ophthalmic research with satisfactory results [25,26]. Because of this, we focused on analyzing tear samples that are relatively easy to collect, store, and process, to identify specific miRNAs (and its genetic targets) that differentially express themselves in the clinically silent interface between OHT and glaucoma, and to support their presumable interest as diagnostic biomarkers for individuals at risk of glaucoma ND. 

## 2. Materials and Methods

We performed an analytical, observational, case-control study including 42 patients recruited from the ophthalmological department of the University Hospital Dr. Peset (Valencia, Spain), who agreed to participate in the study and signed the informed consent. Sample size calculation was performed using the ssize.fdr R package (R Core Team, Vienna, Austria) to detect a 1.5-fold change and achieve an 80% statistical power with a false discovery rate of 15% and an estimated proportion of non-differentially expressed miRNAs of 0.85. The study adhered the Declaration of Helsinki (Edinburgh, 2000) and the Ethics Committee standards of the study center (no. 81/16). All requirements for clinical research to maintain the privacy of the data obtained were met. Two ophthalmologists from the glaucoma section performed a systematized examination of the suitable study participants to ensure their appropriated status (Table 1), which were distributed into two groups: (1) patients diagnosed of POAG (n = 20), and (2) patients with OHT (n = 22).

Ocular examination included the IOP measurement by Goldman applanation tonometry, morphological [ocular fundus by slit-lamp (IMAGEnet, Topcon Barcelona, Spain), and optical coherence tomography (OCT), and Cirrus Spectral domain OCT (Carl Zeiss Meditec, Inc., Madrid, Spain)], and functional [visual field performance, using the 24-2 Swedish interactive threshold algorithm (Humphrey field analyzer, Carl Zeiss Meditec, Inc., Madrid, Spain)] tests. Classification of the glaucoma staging was done according to the descriptions of Mills et al. [27]. 

Sampling was done by collecting reflex tears from the inferior meniscus of the eye without instilling anesthetics, as described in our previous work [25,26], using microhematocrite capillary tube, that were appropriately labeled, and immediately transferred into microcentrifuge tubes and stored in an ultra-freezer at −80 °C. On the day of processing, samples were defrosted and prepared for RNA extraction using the miRCURY RNA Isolation Kit-Biofluids (EXIQON Inc., Woburn, MA, USA). This kit is designed to isolate all RNAs sized less than 1000 nucleotides, from mRNA and tRNA to microRNA and small interfering RNA. We carried out the RNA extraction according to the manufacturer’s instructions. Briefly, the purification is based on spin column chromatography using a proprietary resin as the separation matrix. Small RNAs are separated from other cellular components such as proteins without the use of phenol or chloroform.

The quality and quantity of total RNA obtained from tears was assessed using a Bioanalyzer 2100 (Agilent^®^ Technologies, Inc., Santa Clara, CA, USA), and the RNA 6000 Nano Kit (Agilent^®^ Technologies, Inc.). RNA libraries were prepared using NEBNext^®^ Multiplex Small RNA Library Prep Set for Illumina^®^ (#E7300 y #7580; New England BioLabs^®^, Inc., Ipswich, MA, USA), according to the manufacturer’s protocol https://international.neb.com/protocols/2018/03/27/protocol-for-use-with-nebnext-small-rna-library-prep-set-for-illumina-e7300-e7580-e7560-e7330 (accessed on 17 May 2021). According to the guidelines for low RNA concentration samples, the adapters and RT primers were diluted 1:2 with nuclease-free water and 15 cycles were used for the amplification by PCR. The indexed libraries were purified using the QIAquick^®^ PCR Purification Kit (#28104, QIAGEN^®^, Hilden, Germany). Library quality control was assessed using a 4200 TapeStation (Agilent^®^ Technologies, Inc.) and High Sensitivity D1000 Kit (Agilent^®^ Technologies, Inc.). The miRNA fraction of each library (120–200 bp) was collected using the Pippin Prep System (Sage Science, Inc., Beverly, MA, USA) following the manufacturer’s guidelines and using 3% agarose dye free gel cassettes with internal standards (Marker P) (Sage Science # CDP3010). miRNAs were quantified using a 4200 TapeStation (Agilent^®^ Technologies, Inc.) and High Sensitivity D1000 Kit (Agilent^®^ Technologies, Inc.) prior to normalization and pooling. Sequencing was performed on a NextSeq 500 System (Illumina, Inc., San Diego, CA, USA) with a Mid-Output flow cell for 150-cycle reads obtaining about 3.5 million reads per sample. FASTQ file quality was assessed using FASTQC tool (https://www.bioinformatics.babraham.ac.uk/projects/fastqc/, accessed on 21 May 2021). Adapters and low liability reads were removed. Non-coding RNAs previously described in the ENSEMBL database were selected and characterized. Statistical analyses (normalization, differential expression, and significance) were performed using Limma and edgeR packages deposited in Bioconductor (www.bioconductor.org). A predictive analysis based on receiver operating characteristic (ROC) curves was performed to select those miRNAs showing an area under the curve (AUC) greater than 0.75. Subsequently, an analysis of the main components (PCA) was performed. Later, the target genes of the selected miRNAs were determined using the R miRFA pipeline [28], which is supported by data from the DIANA-TarBase v7, DIANA-microT-CDS and TargetScan v7.1 databases. Additionally, UniProt and Gene Ontology databases have been used to search for terms associated with the trabecular meshwork functions and the glaucoma pathology [29]. To select all genes needed to build a network of metabolic pathways, we considered only those genes that appeared in at least four of the following databases: DisGeNet, GeneDistiller, MalaCards, OMIM PCAN, UniProt, and GO [30]. These genes also need to have at least seven terms in any of the UniProt categories above-mentioned. Two approaches have been used to build the networks: (1) enrichment of metabolic pathways using the g: Profiler, GSEA, Cytoscape, and EnrichmentMap tools [31], and (2) gene function prediction using the GeneMANIA gene integration tool [32], which can function both as an independent server and as an application in Cytoscape. 

For statistical analysis, comparison of two categorical variables was performed using the Pearson Chi square test. We used the Shapiro–Wilk test to check the distribution quantitative variables. The comparison of two means was analyzed by means of the Student t-test for independent samples (normal variables) or the Mann–Whitney U test (non-normal variables). The comparison of more than two means was carried out by the analysis of variance (ANOVA, normal variables) or the Kruskal–Wallis test (non-normal variables). Statistical proceedings were performed using the statistical package ((BM SPSS Statistics for Windows, Version 24.0. Armonk, NY, USA: IBM Corp). 

## 3. Results

### 3.1. Sociodemographic and Ophthalmologic Data

Mean age of participants was 64.5 ± 1.4 years for the POAG group and 61.1 ± 2.4 years for the OHT group. Comparison between groups did not show statistically significant differences (*p* > 0.05). 

Regarding the distribution by gender, both groups showed a greater proportion of women. No statistically significant differences were observed between groups (*p* > 0.05).

Sociodemographic characteristics of the study participants are listed in Table 2. No significant differences were found in any of the study variables. 

The OHT subjects showed elevated IOP but no damage at OCT examination, normal visual fields, and normal ocular fundus. However, POAG patients showed IOP elevation, an increase of optic disc excavation, optic nerve damage, and/or altered visual fields. Ophthalmologic parameters of the study participants are shown in Table 3.

### 3.2. miRNA Identification

**Figure 1:**
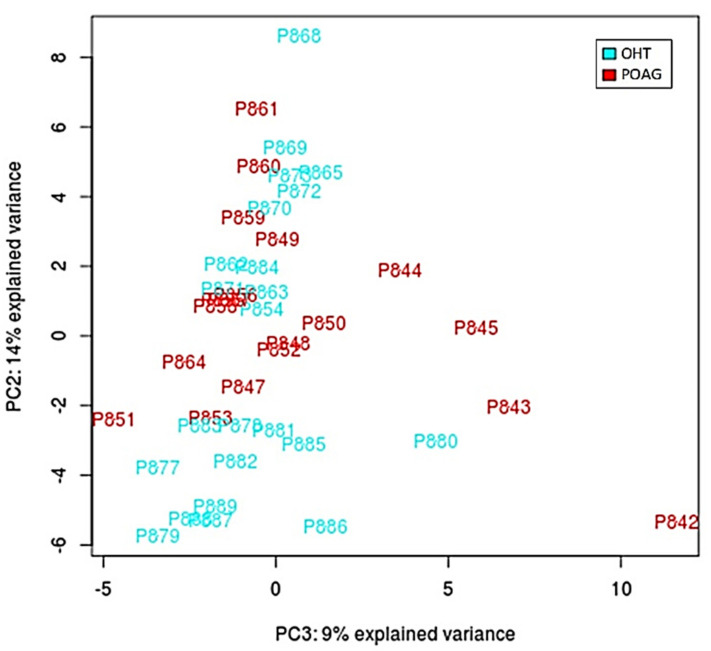
Principal component analysis (PCA) plot after normalization of the data according to the eight differentially expressed miRNAs. Image shows ocular hypertension/ocular hypertension patients (blue marks) and primary open-angle glaucoma (red marks) patients.

After performing all experimental procedures, we were able to identify 95 miRNAs present in tears of OHT and/or POAG patients. Figure 1 shows the PCA plot after the normalization of data. As this figure shows, there was not a good separation between the two groups (POAG in red and OHT in blue) based on the identified miRNAs. 

**Figure 2:**
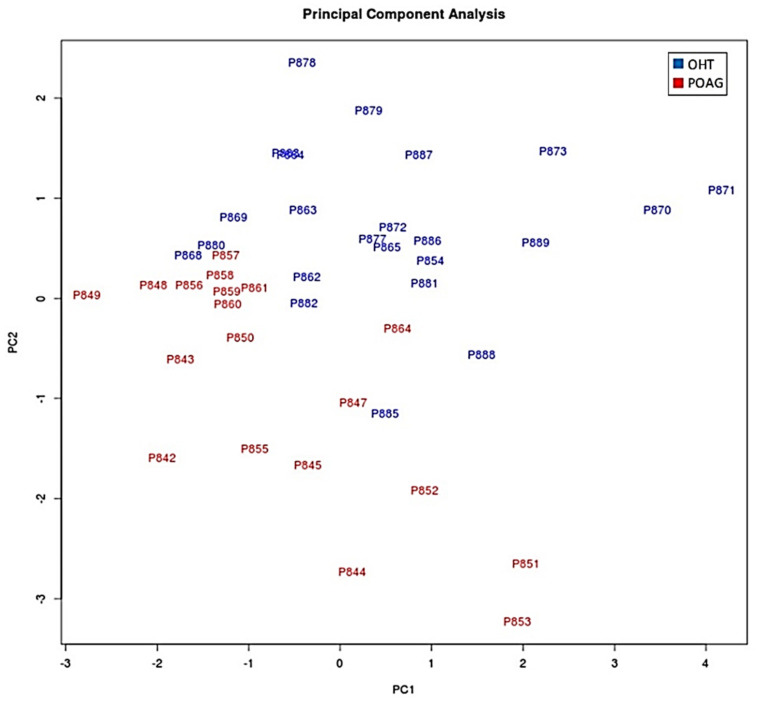
Principal component analysis (PCA) plot comparing the POAG and OHT groups using only miRNAs with statistically significant expression. Red: patients with OHT. Blue: patients with POAG. OHT: ocular hypertension/ocular hypertensives; POAG: primary open angle glaucoma.

The next step was to compare the expression levels of those 95 miRNAs in both study groups (POAG patients vs. OHT subjects). From these 95 miRNAs, 87 showed no significant differences between groups (*p* > 0.05). However, we found six upregulated and two downregulated miRNAs in tears from the POAG patients (Table 4). At this point, we build again the PCA plot, taking into consideration only those eight miRNAs that differentially expressed between groups. The PCA showed a better separation of the miRNAs present in tears from the OHT vs. POAG patients (Figure 2).

In addition, a ROC curve was performed to analyze whether these eight miRNAs could predict POAG in the HTO individuals. Table 5 shows the AUC for each miRNA. The AUC of four of these miRNAs was greater than 0.75, so they could be considered as good predictors of POAG.

### 3.3. Bioinformatic Processing 

Using the databases mentioned in the methodology section and combining their predictions, we identified a total of 14,379 potential target genes of the eight miRNAs. Then, these 14,379 genes were searched in four databases (DisGeNet, GeneDistiller, MalaCards, and OMIM PCAN) that collected information on the association between genes and pathologies, finding the presence of 390, 183, 145, and 7 genes in each of them, respectively. Eleven categories related to trabecular meshwork functions and the glaucoma pathology were selected and the number of genes that had terms in each of them are shown in Table 6. The biological functions marked in bold represent those with the highest number of genes (extracellular matrix and apoptosis processes). In addition, genes related to neurodegeneration, inflammation, and oxidative stress, among others, were also identified.

We performed the Gene Ontology (GO) Biological Process analysis and found 201 items to which the eight identified miRNAs were significantly associated. Table 7 shows the top 40 biological processes of the above miRNAs. In addition to this GO analysis, we identified 36 reactomes (REACT) significantly associated with the eight miRNAs (Table 8), among them, genes related to apoptosis and extracellular matrix conditions. Additionally, those genes involved in aqueous humor homeostasis, oxidative stress, immune response regulation (TGF-β) and neurodegeneration were identified.

**Figure 3:**
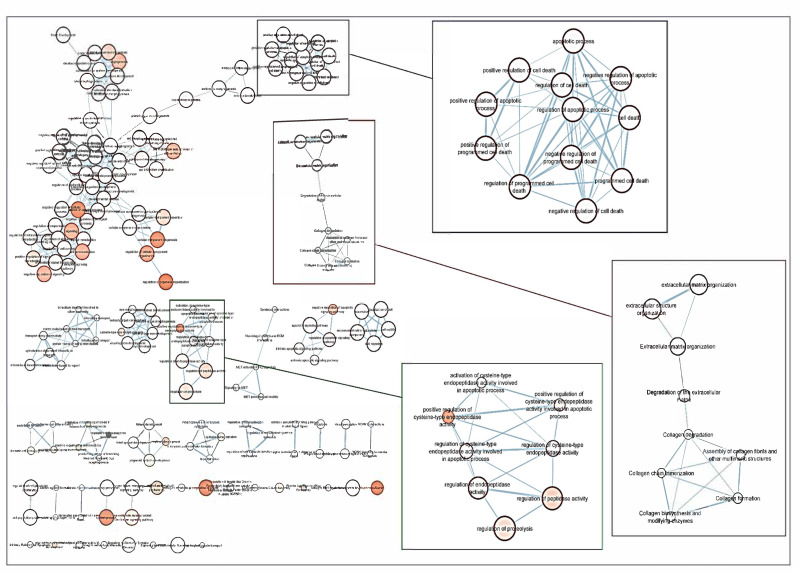
Network of GO terms and Reactome pathways generated with g:Profiler and EnrichmentMap in Cytoscape. Each node (circle) represents a gene set characterized by a particular GO term or reactome pathway. Node fill indicates the enrichment score (FDR q-value). The thickness of blue lines (edges) indicates the number of shared genes (overlap) between two connected nodes. Nodes with high overlap are clustered together, forming groups characterized by similar terms and pathways. Gene clusters involved in apoptosis (blue box), extracellular matrix and collagen concerns (red box), endopeptidase activity (green box) as well as in development pathways (upper left side) have been marked.

Following the screening criteria detailed in the methodology section, a total of 114 genes were selected to build a network of metabolic pathways (Figure 3). This network shows the clusters involved in apoptosis (blue box), extracellular matrix and collagen concerns (red box), endopeptidase activity (green box) as well as in development pathways (upper left side) among other clusters. Network data are included as Supplementary Materials, as both CYS (Cytoscape session file) and SIF (simple interaction file) formats.

By using the GeneMania program, different maps were created. In these maps, each circle is a node representing a gene. Black nodes belong to the original set of 114 genes studied, while grey circles represent those not present as genes in the original set, but very closely related to them according to the selected network criteria. These diagrams also give information about the number of interactions of each gene (the size of the circle is proportional to this number), and the edges indicate a relationship between genes, meaning that the thicker the line, the more intense the relationship. 

**Figure 4:**
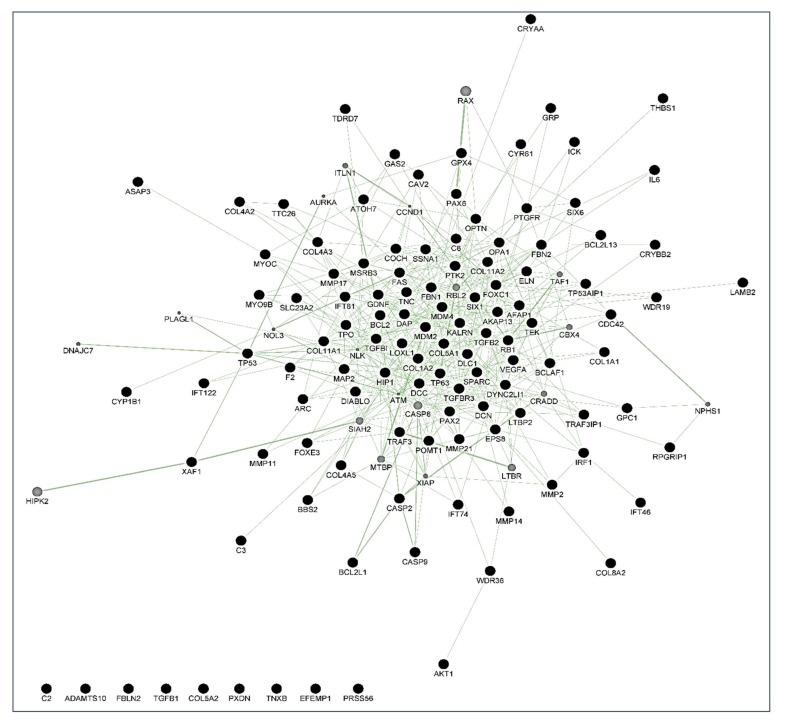
Genetic interaction map created with GeneMania. Black nodes are genes present in the original gene list, grey nodes are genes that are initially not present, but closely related to them. Among the genes, MYOC, OPTN, FOXC1, TGFβ, FAS/TNFR, TP53, CASPs, BCL2, MMPs, and DIABLO were identified. Lines (edges) connecting two genes indicate that they are functionally associated (the effects of perturbing one gene are found to be modified by perturbations to the second gene) using data primary collected from BioGRID. Thicker edges indicate a stronger association.

**Figure 5:**
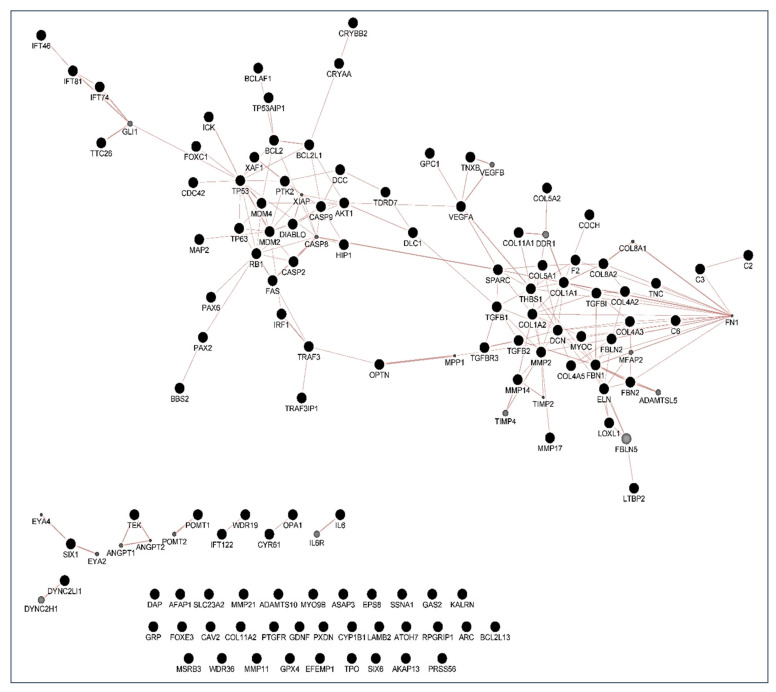
Physical interaction map created with GeneMania. Black nodes are genes present in the original gene list, grey nodes are additional genes that are found interacting with the original ones in a protein–protein interaction study. The genes FOXC1, CASPs, MAP2, TP53, BCL2, DIABLO, IL6, TGFβ, OPTN, MMPs, among others, were identified. Size of grey nodes indicate the likelihood of physical interaction (score), and thicker lines (edges) indicate a stronger interaction. Data are collected from primary studies from protein interaction databases including BioGRID and PathwayCommons.

**Figure 6:**
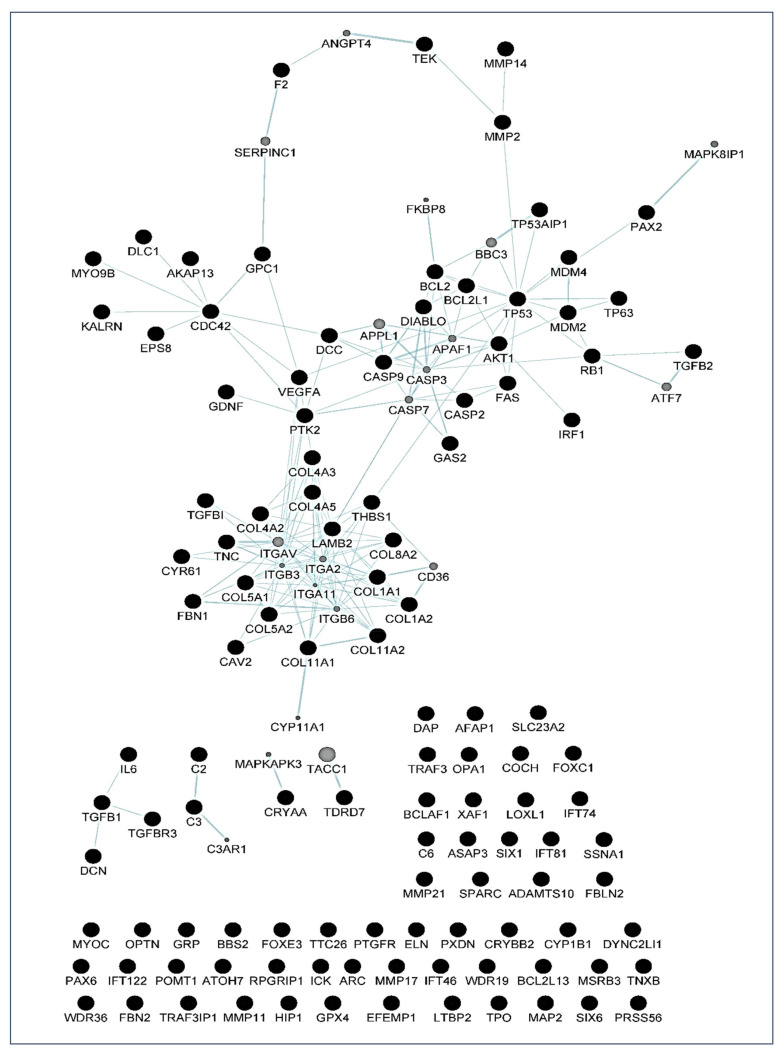
Metabolic pathway map created with GeneMania. Two gene products are linked if they participate in the same reaction within a pathway. Size of gray nodes indicate the likelihood of the gene belonging to the pathway (score), and thicker lines (edges) indicate a stronger relation. Data are collected from various sources such as Reactome and BioCyc, via PathwayCommons.

With these proceedings, we finally built three maps: the first is a map of genetic interactions between these genes (Figure 4); the second is a map showing the physical interactions (Figure 5); and the third is a map of the metabolic pathways (Figure 6). In these figures, grey circles represent genes not present in the original 114 set, but closely related to them. Circle size is proportional to the number of interactions of each gene. Edges indicate a relationship between genes(a thicker line the more intense relationship). Network data from Figure 4, Figure 5 and Figure 6 are also included in the Supplementary Materials as both CYS (Cytoscape session file) and SIF (simple interaction file) formats.

## 4. Discussion

In this work, we successfully profiled the tear miRNAs signature from patients with POAG compared to the OHT, as follows: the miR-26b-5p, miR-152-3p, miR-30e-5p, miR-125b-2-5p, miR-224-5p, miR-151a-3p, miR-1307-3p, and the miR-27a-3p. As far as we know, this is the first study in identifying miRNAs in tears from individuals with HTO and glaucoma patients, revealing its potential as molecular-genetic biomarkers of glaucoma suspect.

People more predisposed to glaucoma must be early identified to undertake measures for avoiding optic nerve irreversible damage and vision-threatening consequences [1,2,3,4,5,6]. Over the years, this concern has continued to shape the thinking of ophthalmologists and researchers worldwide because hypotensive treatment has to be promptly initiated to appropriately control the elevated IOP. In this context, biomarkers might be key diagnostic tools to identify individuals at higher risk for glaucoma such as HTO individuals, just like we did in this report. Our differential expression analysis carried out by NGS led us to identify the tear fingerprint of eight miRNAs that significantly expressed between the POAG patients and HTO individuals. Then, we comprehensively explain the above miRNAs as well as the underlying miRNA-related pathways involved in HTO and POAG, as reflected in Figure 3, Figure 4, Figure 5 and Figure 6 as well as in the Supplementary Materials. 

Specific miRNAs involved in IOP homeostasis and glaucomatous OND were reported by different authors [21,22,23,24]. Others have also conducted similar studies to ours in different ophthalmic disorders [33] using different biological samples [34,35,36]. Liu et al. [37] analyzed the miRNA signature in the aqueous humor of POAG patients and its role in the etiopathogenic mechanisms of glaucomatous OND, reporting 88 miRNAs that differentially expressed between POAG and cataracts. Among them, the miR-151-a-3p was identified in the aqueous humor by these authors, in a similar manner to us in the course of the present work, in which the same miRNA was downregulated in tears of our study participants. Ertekin et al. [38] found miR-26b-5p in plasma from patients with wet macular degeneration. The authors associated this miRNA with oxidative stress occurring in the retinal pigment epithelial cells of the eyes with this disease. We also found the miR-26b-5p in tears of our GPAA patients and OHT individuals. Oxidative/nitrosative stress plays a pivotal role in POAG pathogenesis [12,15,39]; we may hypothesize that in the course of glaucoma, the pro-oxidants may induce the expression of miR-26b-5p, as reflected in this work [39,40,41]. Interestingly, hsa-miR-26b-5p has recently been involved in pseudoexfoliative glaucoma through interaction with the TGFR1 and TGR2 part of SMAD2 [42].

We also described herein that the miRN-27-a-3p showed a significant differential expression in tears from the POAG patients and the OHT participants. This miRNA has been implicated in cell proliferation and apoptosis in different types of cancer [43,44]. Overexpression of this miRNA promotes proliferation and inhibits apoptosis of cancer cells. Likewise, it has been shown that miR27a-3p induces ischemia-reperfusion injury by triggering oxidative stress [45]. In previous reports, it has been shown that the expression of this miRNA was increased in stretched versus control TM cells [46,47]. Consistent with these findings, we suggest that the miR-27a-3p overexpression in tears from POAG patients may be related to both the apoptotic and oxidative stress processes occurring in the POAG eyes [39,40,41]. 

Most target genes corresponding to the eight miRNAs showing significant differential expression profile in tears of the OHT individuals and POAG patients were related to extracellular matrix concerns and apoptosis processes (see the Table 6, Table 7 and Table 8).

Desjarlais et al. [48] reported the upregulation of miR-152-3p in the choroid during the vasodegenerative phase of an oxygen-induced retinopathy model compared to the control animals. However, these authors did not delve into the role of this miRNA, but just described the differential expression profile between groups. However, Wang et al. [49] pointed out that miR-152-3p may be an interesting molecular target for keloid treatment as a relevant regulator of cell proliferation, invasion, and extracellular matrix expression through targeting FOXF1 in keloid fibroblasts [49]. In this regard, we found the miR-152-3p upregulation in tears from the POAG group, supporting data previously reported by other researchers, emphasizing the importance of this miRNA in the trabecular meshwork changes and elevated IOP occurring in OHT and POAG [47].

The miR-30e-5p was differentially expressed in tears from OHT and POAG participants during the present work. However, only one article dealt with its role at the ocular level, highlighting its possible function as a predictor of the myasthenia gravis through the main ocular manifestation of the disease, the blepharoptosis [50]. No publications on the relationship between miR-30e-50 and glaucoma and/or other degenerative eye diseases have been found. Meng et al. [51] reported significant differential expression of the miR-30e-5p in patients with Alzheimer’s disease compared to healthy controls. Additionally, Hardeland et al. [52] reported a negative connection between melatonin availability and neurodegenerative disorders, and in this regard, Scuderi et al. [53] reported that melatonin could protect the eye tissues from free radicals and pro-inflammatory mediators. Taking all these reports into consideration, we may hypothesize that miR-30e-5p may play a role in the ethiopathogenic mechanisms of glaucoma by its negative connection with melatonin. 

Toro et al. [54] carried out a recent study in the vitreous humor of patients with different degrees of diabetic proliferative vitreoretinopathy (PVR) in which six miRNAs whose expression significantly increased with disease progression were reported, among them miR-224-5p. Dysregulation of its expression in PVR patients suggests a possible association with certain retinal degenerative processes. In the present study, we also found the upregulation of this miRNA in the POAG patients, pointing to a potential involvement of miR-224-5p in the onset and/or progression of the glaucoma ND. 

The miR-151a-3p seems to be associated with a high risk of uveal melanoma [55]. Bianciotto et al. [56] reported that OHT is a risk factor for the development of uveal melanoma. In our OHT participants, a significantly higher expression of this miRNA was seen, suggesting that its downregulation in the POAG patients may be related to the onset of glaucoma ND. Nevertheless, the function of this miRNA in glaucoma needs further investigation. 

Oxidative stress, inflammation, and apoptosis are relevant mechanisms involved in POAG [3,4,5,6,7,8,12,15,39]. In this regard, oxidative stress can trigger a wide spectrum of transcription factors such as p53, Nrf2, NF-κB, AP-1, PPAR-γ, and β-catenin/Wnt, among others [57,58,59,60]. When the above molecules undergo activation, they can induce the expression of hundreds of genes such as those related to pro-inflammatory cytokines and chemokines, cell adhesion molecules, metalloproteinases, cell cycle regulators, pro-apoptotic molecules, etc. [60,61,62,63,64]. In the case that the oxidative/inflammatory environment will be prolonged, this can chronically affect the ocular cells and tissues leading to apoptotic cell death and glaucoma ND. Given the target genes of the eight miRNAs with different expression between POAG and OHT, and following the methods explained above, we conducted a bioinformatic work to build several maps that showed the interactions between these genes as well as a connection network between the different metabolic pathways associated with these genes and miRNAs, as reflected in Figure 3, Figure 4, Figure 5 and Figure 6. Target genes of the eight miRNAs that were identified in tears such as those showing a significant differential expression profile between the OHT and the POAG groups were mainly related to apoptosis and extracellular matrix functions as well as aqueous humor homeostasis, focal adhesion, oxidative stress, inflammation signaling and ND, as reflected in Figure 3, Figure 4, Figure 5 and Figure 6. 

Up to today, genetic testing can help identify people at risk for early-onset glaucoma types in almost 30% of cases [65]. A useful gene-based screening test for adult-onset POAG forms is not yet available [66], but a new test in blood or saliva by multi-trait analyses have found the risk of a person developing glaucoma [67]. Nevertheless, no way exists to predict the risk of a person with OHT to develop glaucoma. 

We propose that the miRNAs identified in the present work and their target genes/signaling pathways will allow for a better understanding of the molecular and genetic bases of glaucoma and, in this way, gain knowledge that leads us to develop a better diagnosis strategy for properly identifying HTO at a higher risk of glaucoma ND.

Individuals predisposed to glaucoma such as those suffering OHT should be closely followed to identify early changes to promptly start hypotensive therapy. Top advances in glaucoma genetics have provided an open window for stratifying the risk of glaucoma in HTO cases based on molecular-genetic predictions. Here, we show for the first time a new approach and future avenues dealing with miRNA expression in tears to better diagnose HTO individuals and identify those at highest risk of POAG as well as to better eye/vision care and blindness prevention.

## 5. Conclusions

In summary, eight miRNAs: **the miR-26b-5p, miR-152-3p, miR-30e-5p, miR-125b-2-5p, miR-224-5p, miR-151a-3p, miR-1307-3p and the miR-27a-3p** were found to be differentially expressed in the tears of patients in the interface between OHT and POAG. We demonstrated that some of these miRNAs, their target genes, and signaling pathways have already been related to **apoptosis, extracellular matrix functions, inflammation, and oxidative stress as well as aqueous humor homeostasis and neurodegeneration.** Further studies are needed to validate our results and deepen the knowledge of these miRNAs to discern its potential as biomarkers for the diagnosis and/or prognosis and future biotherapies for OHT and glaucomatous NDG.

## Figures and Tables

**Table 1:** Inclusion and exclusion criteria for the study participants.

INCLUSION CRITERIA	
POAG Group	OHT Group
Diagnosis of POAG	OHT without signs of neurodegeneration
Ranging 50–80 years	
Both genders	
Capacity to understand and participate in the study	
**EXCLUSION CRITERIA**	
**POAG Group**	**OHT Group**
Other GLs different from POAG	With signs of glaucoma neurodegeneration
<40 or >80 years old	
Other ocular diseases or systemic pathologies that may interfere with the study	
Other treatments that may interfere with the study results	
Ocular surgery or laser treatment during the last year	
Contact lenses wearing	
Unable to participate in the study	

POAG: primary open angle glaucoma; OHT: ocular hypertension GL: glaucoma.

**Table 2:** Sociodemographic characteristics of the study participants.

Variables		POAG	OHT	*p* *
**Age (years)**		64.5 (1.4)	61.1 (2.4)	0.218
**Gender (%, men/women)**		47.6/52.4	33.3/66.7	0.366
**Height (cm)**		165.2 (2.2)	164.0 (2.2)	0.694
**Weight (kg)**		76.7 (3.7)	70.6 (3.2)	0.217
**BMI (kg/m^2^)**		28.1 (1.2)	26.2 (0.9)	0.238
**Smoking (%)**		28.6	22.2	0.651
**Alcohol consumption (%)**		9.5	16.7	0.506
**Physical activity (%)**	**Mild**	42.9	61.1	0.225
	**Moderate**	57.1	33.3	
	**High**	0.0	5.6	

POAG: primary open-angle glaucoma; OHT: ocular hypertension; BMI: body mass index. Data shows the mean (standard deviation) or percentage. * Significance level was set at 0.05.

**Table 3:** Ophthalmological parameters of the study participants.

Variables	POAG	OHT	* p Value
**BCVA (decimal)**	0.86 ± 0.03	0.90 ± 0.03	0.382
**IOP (mmHg)**	16.00 ± 0.58	18.17 ± 0.81	0.033
**C-D ratio**	0.46 ± 0.02	0.046 ± 0.05	0.949
**CCT**	536.86 ± 6.56	554.42 ± 8.12	0.141
**VF-PSD**	2.27 ± 0.29	1.33 ± 0.13	0.008
**VFI**	90.57 ± 2.30	92.22 ± 1.09	0.543
**VF-MD**	−0.56 ± 0.91	0.98 ± 0.410	0.154
**OCT-papillary excavation**	0.61 ± 0.04	0.057 ± 0.04	0.439
**OCT-fibrillar thickness**	84.71 ± 3.13	88.61 ± 2.29	0.334
**OCT-rim area**	4.58 ± 2.29	1.11 ± 0.05	0.169
**RGCs density**	68.95 ± 2.23	77.89 ± 2.15	0.008

POAG: primary open-angle glaucoma; OHT: ocular hypertension; BCVA: best corrected visual acuity; IOP: intraocular pressure; C-D: cup-to-disc; CCT: central corneal thickness; VF: visual field; PSD: pattern standard deviation; VFI: visual field index; MD: mean deviation; OCT: optical coherence tomography; RGCs: retinal ganglion cells. * Statistical significance (*p* < 0.05).

**Table 4:** Differences in miRNA expression between groups.

miRNA ID	Fold Change (POAG vs. OHT) ^§^	*p* *
**hsa-miR-26b-5p**	0.855	0.012
**hsa-miR-27a-3p**	0.774	0.004
**hsa-miR-152-3p**	0.753	0.004
**hsa-miR-30e-5p**	0.901	0.005
**hsa-miR-125b-2-5p**	0.529	0.027
**hsa-miR-224-5p**	0.745	0.033
**hsa-miR-151a-3p**	−0.417	0.009
**hsa-miR-1307-3p**	−1	0.004

POAG: primary open-angle glaucoma; OHT: ocular hypertension. ^§^ OHT was the reference group. * Significance was set at 0.05.

**Table 5:** Area under the curve for the eight miRNAs with significant differential expression between groups.

miRNA	AUC
**hsa-miR-26b-5p**	0.81693
**hsa-miR-152-3p**	0.75743
**hsa-miR-30e-5p**	0.76201
**hsa-miR-125b-2-5p**	0.67276
**hsa-miR-224-5p**	0.72082
**hsa-miR-151a-3p**	0.75972
**hsa-miR-1307-3p**	0.69565
**hsa-miR-27a-3p**	0.68192

POAG: primary open-angle glaucoma; OHT: ocular hypertension/hypertensives; AUC: area under the curve.

**Table 6:** Target genes of the eight miRNAs showing significantly different expression profile between groups.

Biological Function	Number of Genes
IOP, aqueous humor outflow	420
**Extracellular matrix**	**1143**
Ciliary body functions	593
Focal adhesion	558
Oxidative stress response	272
**Apoptosis**	**1516**
Neurodegeneration	31
Retinal ganglion cells	27
RHO signaling	213
TGF-β signaling	5
Eye development	420

TM: trabecular meshwork; IOP: intraocular pressure; RHO: Ras homologous protein family; TGFβ: transforming growth factor beta.

**Table 7:** Top 40 GO Biological Processes associated to POAG and with target miRNAs.

	ID	Name	N	*p*-Value		ID	Name	N	*p*-Value
1	GO:0030198	extracellular matrix organization	31	4.63E-25	21	GO:0016043	cellular component organization	75	1.88E-09
2	GO:0043062	extracellular structure organization	31	5.15E-25	22	GO:0008219	cell death	40	1.89E-09
3	GO:0009653	anatomical structure morphogenesis	51	2.36E-15	23	GO:0071840	cellular component organization or biogenesis	76	2.58E-09
4	GO:0150063	visual system development	19	7.78E-15	24	GO:0048592	eye morphogenesis	11	3.22E-09
5	GO:0048880	sensory system development	19	1.36E-14	25	GO:0042981	regulation of apoptosis	33	3.92E-09
6	GO:0001654	eye development	18	1.20E-13	26	GO:0012501	programmed cell death	38	5.16E-09
7	GO:0048513	animal organ development	52	1.29E-13	27	GO:0090596	sensory organ morphogenesis	12	7.97E-09
8	GO:0009887	animal organ morphogenesis	29	2.37E-13	28	GO:0009888	tissue development	35	1.41E-08
9	GO:0007423	sensory organ development	19	1.27E-12	29	GO:0050793	regulation of development	42	2.22E-08
10	GO:0007275	multicellular organism development	65	3.25E-12	30	GO:0072359	circulatory system development	26	4.96E-08
11	GO:0048731	system development	61	3.20E-11	31	GO:0007166	cell surface receptor signaling pathway	48	7.24E-08
12	GO:0030199	collagen fibril organization	10	1.50E-10	32	GO:0042073	intraciliary transport	9	8.63E-08
13	GO:0048646	morphogenesis	29	1.74E-10	33	GO:0097190	apoptotic signaling pathway	20	1.02E-07
14	GO:0032502	development	70	3.01E-10	34	GO:0032501	multicellular organismal process	73	1.22E-07
15	GO:0010941	regulation of cell death	36	4.22E-10	35	GO:0007601	visual perception	12	1.78E-07
16	GO:0043010	anterior chamber eye development	14	4.33E-10	36	GO:0010970	transport along microtubule	12	1.95E-07
17	GO:0048856	anatomical structure development	66	5.22E-10	37	GO:0050953	sensory perception of light stimulus	12	3.29E-07
18	GO:0006915	Apoptosis	37	1.24E-09	38	GO:0032963	collagen metabolic process	10	3.95E-07
19	GO:0043067	Apoptosis regulation	34	1.33E-09	39	GO:0035735	intraciliary transport involved in cilium assembly	8	4.31E-07
20	GO:0006928	movement of cell or subcellular component	42	1.75E-09	40	GO:0098840	protein transport along microtubule	9	4.81E-07

GO: Gene Ontology; POAG: primary open-angle glaucoma; N: number of genes.

**Table 8:** Reactomes associated with the 8 tear differentially expressed miRNAs.

ID	Name	N	*p*-Value
REAC:R-HSA-1474244	Extracellular matrix organization	29	5.16E-21
REAC:R-HSA-1474228	Extracellular matrix degradation	18	2.59E-14
REAC:R-HSA-1442490	Collagen degradation	13	1.48E-12
REAC:R-HSA-3000178	ECM proteoglycans	13	1.33E-11
REAC:R-HSA-3000171	Non-integrin membrane-ECM interactions	12	1.43E-11
REAC:R-HSA-2022090	Assembly of collagen fibrils (and other multimeric structures)	12	2.21E-11
REAC:R-HSA-8948216	Collagen chain trimerization	10	7.82E-10
REAC:R-HSA-1474290	Collagen formation	12	3.08E-09
REAC:R-HSA-2243919	Crosslinking of collagen fibrils	7	2.62E-08
REAC:R-HSA-216083	Integrin cell surface interactions	11	3.35E-08
REAC:R-HSA-8874081	MET activates PTK2 signaling	8	3.46E-08
REAC:R-HSA-1650814	Collagen biosynthesis and modifying enzymes	10	6.68E-08
REAC:R-HSA-8875878	MET promotes cell motility	8	4.25E-07
REAC:R-HSA-1566948	Elastic fiber formation	8	9.52E-07
REAC:R-HSA-5620924	Intraflagellar transport	8	5.22087E-06
REAC:R-HSA-2129379	Molecules associated with elastic fibers	7	7.44538E-06
REAC:R-HSA-109581	Apoptosis	12	8.49652E-06
REAC:R-HSA-5357801	Apoptosis regulation	12	1.0277E-05
REAC:R-HSA-3000170	Syndecan interactions	6	2.27603E-05
REAC:R-HSA-2214320	Anchoring fibril formation	5	4.00759E-05
REAC:R-HSA-6806834	Signaling by MET	8	8.14274E-05
REAC:R-HSA-109606	Intrinsic Pathway for Apoptosis	7	9.9991E-05
REAC:R-HSA-375165	NCAM signaling for neurite out-growth	7	2.12464E-04
REAC:R-HSA-9006934	Signaling by Receptor Tyrosine Kinases	16	3.8436E-04
REAC:R-HSA-419037	NCAM1 interactions	6	4.6565E-04
REAC:R-HSA-6785807	Interleukin-4 and Interleukin-13 signaling	8	1.40305E-03
REAC:R-HSA-186797	Signaling by PDGF	6	2.114864E-03
REAC:R-HSA-5617833	Cilium Assembly	10	2.548397E-03
REAC:R-HSA-381426	IGF transport & uptake by IGF Binding Proteins (IGFBPs)	8	3.42679E-03
REAC:R-HSA-76009	Platelet Aggregation (Plug Formation)	5	5.752906E-03
REAC:R-HSA-5633008	TP53 Regulates Transcription of Cell Death Genes	5	1.1963399E-02
REAC:R-HSA-76002	Platelet activation, signaling and aggregation	10	2.1334022E-02
REAC:R-HSA-3000157	Laminin interactions	4	4.1196772E-02
REAC:R-HSA-114452	Activation of BH3-only proteins	4	4.1196772E-02
REAC:R-HSA-6803207	TP53 regulation of Caspase activator/Caspases Transcription	3	4.5562937E-02

N: number of genes; REAC: reactome.

## Data Availability

The data is enclosed in the present work. Supplementary Material is also offered through this article. Network data from Figure 4, Figure 5 and Figure 6 are also included in the Supplementary Materials as both CYS (Cytoscape session file) and SIF (simple interaction file) formats.

## Supplementary Materials

The following are available online at https://www.mdpi.com/article/10.3390/jcm10112227/s1. Supplementary Materials includes network data from Figure 3, Figure 4, Figure 5 and Figure 6 as both CYS (Cytoscape session file) and SIF (simple interaction file) formats.
